# Adsorption Capacity of Activated Carbon-Encapsulated Hollow-Type Spherical Bacterial Cellulose Gels for Uremic Toxins in a Simulated Human Gastrointestinal Environment

**DOI:** 10.3390/gels10070417

**Published:** 2024-06-25

**Authors:** Aya Hirai, Masashige Suzuki, Kaito Sato, Toru Hoshi, Takao Aoyagi

**Affiliations:** 1 Department of Materials and Applied Chemistry, Graduate School of Science and Technology, Nihon University, 1-8-14, Kanda-Surugadai, Chiyoda-ku, Tokyo 101-8308, Japan; csay21002@g.nihon-u.ac.jp (A.H.); csma19025@g.nihon-u.ac.jp (M.S.); cska22016@g.nihon-u.ac.jp (K.S.); 2 Department of Materials and Applied Chemistry, College of Science and Technology Nihon University, 1-8-14, Kanda-Surugadai, Chiyoda-ku, Tokyo 101-8308, Japan; aoyagi.takao@nihon-u.ac.jp

**Keywords:** bacteria cellulose gel, activated carbon, seamless capsule, chronic kidney disease

## Abstract

To reduce the risk of the adsorption of granular activated carbon in the gastrointestinal tract, we successfully produced a hollow-type spherical bacterial cellulose (HSBC) gel containing activated carbon with a particle size of 6 μm. In this study, the aim of which was to develop an effective formulation, we evaluated the stability of activated-carbon-encapsulating HSBC gels under various pH conditions. Activated-carbon-encapsulating HSBC gels (ACEGs) retained the activated carbon without leaking when subjected to agitation in acidic or basic environments. The saturated adsorption amount, calculated using the Langmuir adsorption isotherm, was affected by the target adsorbate and pH conditions. These results indicate that ACEGs can adsorb uremic toxins and their precursors similarly to conventional uremic toxin adsorbents while preventing direct contact between the encapsulated activated carbon and the gastrointestinal tract. Compared to powdered activated carbon, the ACEG is less likely to be adsorbed in the gastrointestinal tract. Therefore, the proposed ACEG is a promising new formulation that will contribute to the treatment of renal failure and improve patients’ compliance with medication.

## 1. Introduction

In recent years, measures against chronic kidney disease (CKD) have been widely taken up because of the rapid increase in the number of dialysis patients [1] and the clarification of cardiovascular risk in CKD patients [2]. In general, toxins accumulate in the body due to decreased renal function, which is thought to be one of the causes of uremia symptoms [3]. A variety of uremic toxins have been reported, with indoxyl sulfate recently garnering attention in relation to drug therapy and various pathological conditions [4].

The toxin is produced in the following way: gut bacteria convert tryptophan, derived from food, into indole, which is then metabolized in the liver after intestinal absorption to become indoxyl sulfate. Animal studies have shown that indoxyl sulfate reduces renal filtration ability [5]. The decrease in renal function makes blood indoxyl sulfate difficult to remove. Therefore, a vicious cycle occurs, in which the blood indoxyl sulfate concentration further increases and renal function further decreases as a consequence [6].

Activated-carbon-based adsorbents have been employed in an attempt to remove uremic toxins. Currently, AST-120 (KREMEZIN^®^), a formulation that uses activated carbon, is used to improve uremia symptoms and delay the initiation of dialysis [7]. Kremezin®, an activated-carbon-based adsorbent primarily composed of 200–400 µm spherical adsorbed charcoal, is characterized by poor palatability and difficulty in swallowing. Its daily dosage of 6 g may reduce compliance due to discomfort during administration. Moreover, the adherence of activated carbon to the intestinal tract carries a potential risk of constipation, bowel obstruction, and ulcers [8,9,10]. Activated carbon with a smaller particle size has a larger specific surface area and larger pore volume [11]. However, activated carbon with a smaller particle size is unsuitable for oral administration due to its potential for adhering to the intestinal tract. Additionally, patients with renal failure may have difficulty swallowing powdered activated carbon without discomfort due to their limited fluid intake. Hence, it is crucial to devise a novel dosage formulation that facilitates ingestion, enhances palatability, and minimizes the required dose.

Many research studies have already been published on the encapsulation of activated carbon in agar gel [12] and alginate gel [13]. This approach offers the advantages of an improvement in the ability to handle granular activated carbon and enhanced ease of swallowing. Additionally, encapsulation is expected to avert physical contact between activated carbon and the digestive tract, thereby reducing the risk of adsorption. Nevertheless, concerns have been raised that capsules may rupture in response to pH changes and peristaltic movements, leading to activated carbon leakage. Consequently, the use of a high-strength encapsulation material is necessary to withstand such physiological activity.

In our laboratory, we have succeeded in preparing a hollow-type spherical bacterial cellulose gel (HSBC gel), containing fluorescent particles larger than the pore size of the bacterial cellulose (BC) gelatinous membrane, through the biosynthesis of BC gel on the surface of calcium alginate gel containing fluorescent particles [14]. The BC gelatinous membrane of the HSBC gel surface is composed of a three-dimensional network structure consisting of cellulose fibrils. Substances smaller than the pore size of the mesh can penetrate through the BC gelatinous membrane, allowing the HSBC gel cavity to be filled with various substances. Furthermore, HSBC gel does not permeate substances larger than the pore diameter of the BC gelatinous membrane, allowing for selective permeation based on the size of the substance. These properties make HSBC gel a promising material for applications in the medical field; in particular, it could be used in drug capsules and for immuno-separation.

As stated in our previous report, we have succeeded in preparing a HSBC gel, encapsulating activated carbon, with a particle size of 6 μm. This gel could adsorb and immobilize the uremic toxin in the internal cavity without leakage to the exterior. The gel is not easily decomposed by acids, bases, or enzymes [15], and therefore, it is expected to traverse the gastrointestinal tract while adsorbing uremia-causing substances without leakage into the tract. The aforementioned advantages mean that the gel represents a promising new formulation that reduces the daily dosage and frequency of administration while minimizing the associated risks.

In this study, we evaluated the adsorption capacity of this activated-carbon-encapsulating HSBC gel towards indole and tryptophan, which are known precursors of uremic toxins [5]. To develop an effective formulation, we investigated the maximum adsorption capacity, differences in adsorption capacity depending on the type of toxin precursor, and the effect of ions other than the target substance on the adsorption capacity. Additionally, we assessed the stability of the activated-carbon-encapsulating HSBC gel under varying pH values.

## 2. Results and Discussion

### 2.1. Stability Evaluation of Activated-Carbon-Encapsulating HSBC Gels in Static Conditions

**Figure 1:**
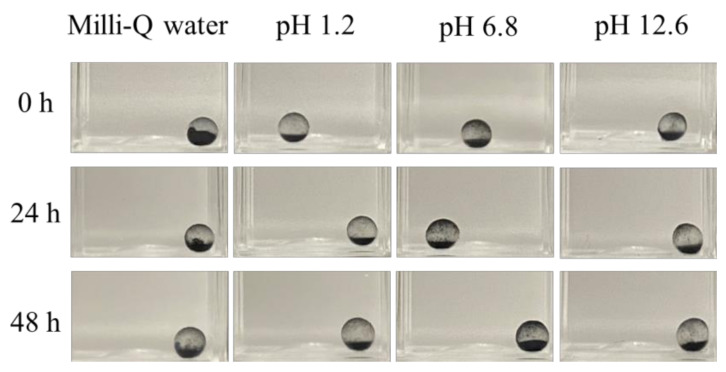
Photographs of ACEGs immersed under different pH and time conditions.

The results of the stability evaluation conducted under static conditions are shown in Figure 1. No leakage of the encapsulated activated carbon from the HSBC gel was observed under any tested conditions. These results indicate that the activated-carbon-encapsulating HSBC gel (ACEG) remained intact for 48 h under pH conditions analogous to those of gastric fluids, intestinal fluids, and more basic conditions, without any release of the encapsulated activated carbon. The ACEG resists degradation by gastric or intestinal fluids and effectively retains the activated carbon without leakage.

### 2.2. Stability Evaluation of ACEGs under Agitation Conditions

**Figure 2:**
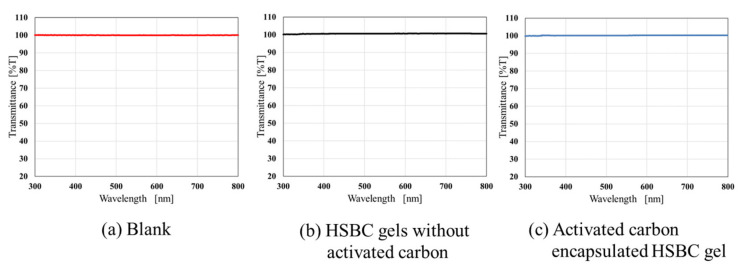
Transmittance of solution after 48 h immersion in Milli-Q water of (**a**) a blank sample, (**b**) HSBC gel without activated carbons, and (**c**) ACEG.

The results, obtained using Milli-Q water, are shown in Figure 2. The transmittances of a blank sample and the HSBC gel without activated carbon were nearly identical, indicating that the HSBC gel does not affect transparency. Similarly, the HSBC gel did not affect light permeability in Solution No. 1 (pH 1.2), Solution No. 2 (pH 6.8), or NaOH aqueous solution (pH 12.6). Additionally, (c) ACEG exhibited a transmittance close to 100%. Therefore, neither the disintegration of ACEG nor the leakage of encapsulated activated carbon was observed in Milli-Q water.

**Figure 3:**
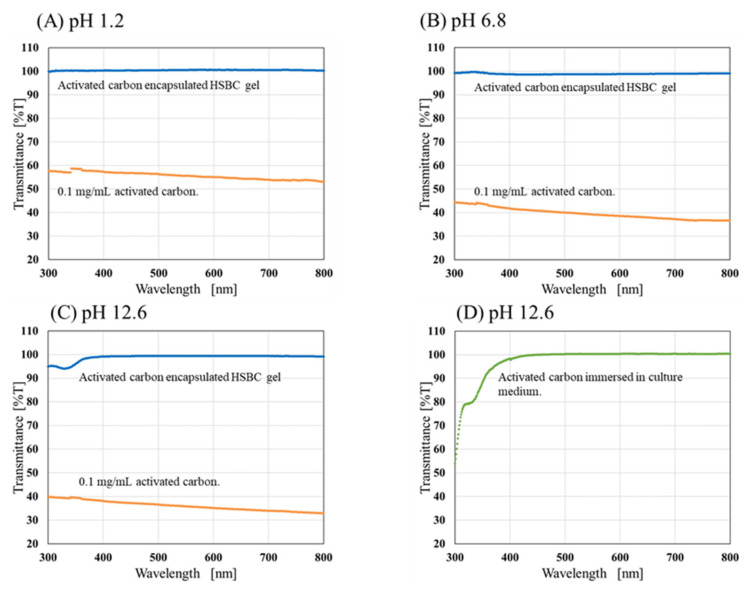
Transmittance of solution of after 48 h immersion of activated-carbon-encapsulating HSBC gel and 0.1 mg/mL activated carbon at different pH conditions. (**A**) Disintegration Test Solution No. 1, pH 1.2; (**B**) Disintegration Test Solution No. 2, pH 6.8; (**C**) NaOH aqueous solution, pH 12.6; and (**D**) activated carbon immersed in culture medium for 24 h was immersed in NaOH aqueous solution (pH 12.6) for 48 h, and the supernatant was obtained by filtration.

The transmittance of ACEG and 0.1 mg/mL activated carbon suspension after 48 h of immersion in test solutions of different pH are shown in Figure 3. Across all pH conditions, the transmittance of the activated carbon suspension decreased across the entire spectrum of measured wavelengths. In contrast, at pH 1.2 (Figure 3A) and pH 6.8 (Figure 3B), the transmittance of the ACEG remained at nearly 100% after 48 h. Therefore, under these conditions, neither the HSBC gel nor the leakage of the encapsulated activated carbon was observed.

At pH 12.6 (Figure 3C), the ACEG exhibited a decline in transmittance at wavelengths below 350 nm. This decline differed from the overall decrease, as in the 0.1 mg/mL activated carbon suspension, indicating that the activated carbon within the HSBC gel was non-leaking. Therefore, the cause of this decrease in transmittance below 330 nm was investigated in the following experiment. The activated carbon underwent immersion in the culture medium for 24 h, followed by immersion in NaOH aqueous solution for 48 h. Subsequently, the activated carbon was filtered using a syringe filter with a pore size of 0.2 µm, and the transmittance of filtrate was measured (Figure 3D). As a result, a similar absorption pattern around 330 nm was observed, indicating that this absorption might originate from a medium component adsorbed during the culture stage. Additionally, no leakage of activated carbon was detected even under pH 12.6 conditions, which are not encountered in the gastrointestinal tract. These results suggest that the collapse of the HSBC gel and the leakage of the encapsulated activated carbon is unlikely to occur under the pH conditions found within the gastrointestinal tract. Therefore, ACEG might be able to pass through the gastrointestinal tract without leaking the activated carbon.

### 2.3. Evaluation of Adsorption Capacity of Uremic Toxins in the Presence of Coexisting Ions

**Figure 4:**
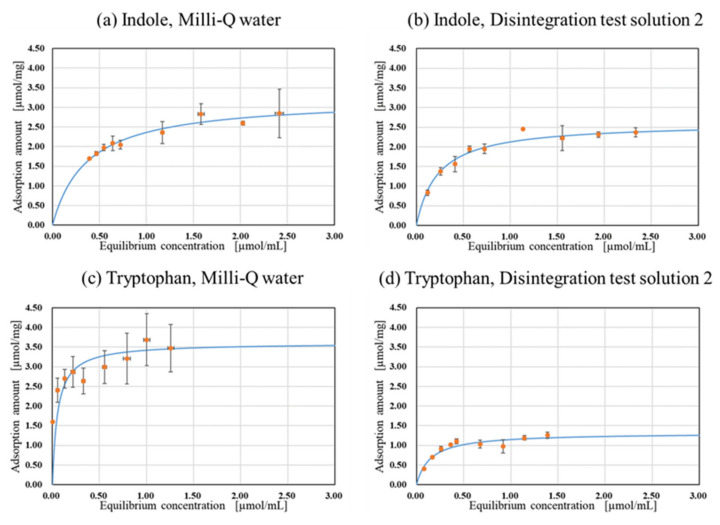
Adsorption isotherms of encapsulated activated carbon. The orange plots are experimental values. The blue line is the curve fitted to the Langmuir equation using the parameters in Table 1. (n ≧ 3).

The relationship between each equilibrium concentration of ACEG and the saturated adsorption amount is shown in Figure 4. Table 1 displays the saturated adsorption levels and the Langmuir constant, as obtained from Equation (3). As seen in Figure 4a–d, under all the conditions, the adsorption of uremic toxin precursors by the ACEG adhered to Langmuir’s adsorption isotherm. In all conditions, *R*^2^ approached 1.0, suggesting that the adsorption of uremic toxin precursors by the ACEG adhered to Langmuir’s adsorption isotherm.

The saturated adsorption levels of indole and tryptophan in Milli-Q water were similar. The adsorption results obtained when utilizing Test Solution No. 2 indicated that the ACEG adsorbed both indole and tryptophan, even in the presence of coexisting ions. To investigate the effect with and without encapsulation, the adsorption behavior of indole when adsorbed onto intact activated carbon in Milli-Q water was studied. Following encapsulation, *Q_m_* was reduced, due to some substrates remaining on the activated carbon from the cultivation that obstructed the adsorption of indole molecules. However, the adsorption level seemed to be satisfactory, meaning that the capsulated formulation should be suitable and practical for clinical use. According to other publications, different kinds of absorbents have been used to investigate the adsorption of uremic toxins, as measured using Langmuir isotherms, and they were successful in estimating saturated adsorption [16,17,18,19,20]. In terms of activated carbon absorbance, Wang, W. et al. showed that porous activated carbon from rubber seed shells effectively removed creatinine and uric acid, and, using Langmuir isotherms, they elucidated that the level of saturated adsorption of creatinine was 3.81 µmol/mg [21]. This is comparable to our data on indole and tryptophan. Recently, Guangle, Q. et al. summarized the adsorption capacity of uremic toxins in comparison with other types of absorbents [22], and the levels found in this study were higher than those found for the absorbents.

Nonetheless, the saturated adsorption amount of tryptophan in Test Solution No. 2 was significantly diminished compared to that of indole. Comparing the pKa of indole and tryptophan, indole exhibits a pKa of −2.4 [23], whereas for tryptophan, pK_1_ = 2.38, pK_2_ = 9.34, and PI = 5.89 [24]. In Test Solution No. 2 (pH 6.8), over 99% of tryptophan exists as a zwitterion. Interactions have been reported to occur between inorganic ions and the zwitterionic forms of amino acids, such as glycine [25] and alanine [26]. Hence, aggregates may have been formed due to the interaction of tryptophan and inorganic ions during disintegration in Test Solution No. 2. Previous studies have reported that substances with larger molecular weights tend to adsorb less onto activated carbon than substances with smaller molecular weights [27]. Furthermore, the coconut shell activated carbon utilized in this study is intended for relatively small molecular sizes [28]. This implies that the molecular size of the aggregates formed between tryptophan zwitterions and coexisting inorganic ions increases, and the amount of adsorption therefore decreases.

The above results suggest that ACEG is capable of adsorbing uremic toxin precursors under in vivo pH conditions without the leakage of activated carbon. However, coexisting inorganic ions may affect the saturated adsorption capacity of activated carbon within the HSBC gel.

Regarding administration by actually swallowing the formulation, hydrogels are good materials for overcoming difficulties such as dysphagia. According to the International Dysphagia Diet Standardisation Initiative (IDDSI), hydrogels from polysaccharides are recommended as practical candidates [29]. A hydrogel from carboxymethylcellulose showed the capacity to improve the swallowing of a relatively large tablet of metformin hydrochloride for diabetes treatment [30]. Therefore, hydrogel-based materials such as the ACEG proposed in this study are practical for clinical use.

In this study, we sought to investigate the removal activities of precursors of uremic toxins from the gastrointestinal tract by means of the adsorption activities of ACEG, because these compounds are causative agents of uremic toxins in the gastrointestinal tract. However, it is interesting to investigate the adsorption behavior of compounds other than indole and tryptophan. Many researchers have discussed other uremic toxins, such as creatine, urea, uric acid, that should be removed from the blood [21,22]. These researchers, through the development of effective absorbents, have contributed to improving blood purification for patients with chronic renal failure.

The ACEG used in this study would also be applicable for the removal of uremic toxins in blood by considering the blood compatibility of cellulose-based materials, including the consideration of clotting and the immune response. A review by Cho et al. summarizes numerous studies on cellulose nanofibers and also discusses the biocompatibility of this material [31]. Its applications include its potential use in wound dressings, scaffolding for cartilage tissue regeneration, bone tissue engineering, and artificial blood vessels and vascular grafts. Although ACEG alone may sometimes require the chemical modification of cellulose or conjugation with other bioactive substances, it is very promising.

Many studies have been published on the chemical modification of nanofibers. Nanofibers have high mechanical strength due to their crystallinity, and if molecules that inhibit thrombus formation, such as heparin, can be immobilized using a small number of hydroxyl groups that do not affect the crystallinity [32], the potential applications in relation to the removal of blood uremic toxins by ACEG will be further enhanced.

## 3. Conclusions

The ACEG remained stable even when agitated in acidic or basic environments. Consequently, as ACEG maintains its structural integrity within the gastrointestinal tract, it is thus anticipated to pass through it without leaking the encapsulated activated carbon. Moreover, the ACEG exhibits the capability to adsorb indole and tryptophan even in the presence of inorganic ions.

These results demonstrate the ACEG’s capacity to adsorb uremic toxins and their precursors while preventing direct contact between the encapsulated activated carbon and the gastrointestinal tract. Compared to powdered activated carbon, the ACEG minimizes the risk of adsorption in the gastrointestinal tract. Consequently, the ACEG is a promising new formulation that contributes to the treatment of renal failure and improves patients’ compliance with medication.

## 4. Materials and Methods

### 4.1. Materials

The medium we used consisted of a mixture of 45.0 g D-glucose (Kanto Chemical Co., Inc., Chou-ku, Tokyo, Japan), 2.5 g mannitol (Kanto Chemical Co., Inc.), 2.5 g polypeptone (HIPOLYPEPTONETM, Nihon Pharmaceutical Co., Ltd., Chou-ku, Tokyo, Japan), 2.5 g Bacto^TM^ yeast extract (BD Biosciences, Franklin Lakes, NJ, USA), and 0.5 g magnesium sulfate heptahydrate (MgSO_4_∙7H_2_O: Kanto Chemical Co., Inc.) in 500 mL Milli-Q water. Silicone oils (KF-56A: 0.995 g/cm^3^, 15 mm/s^2^, ethanol-soluble oil) were obtained from Shin-Etsu Chemical Co., Ltd. (Chiyoda-ku, Tokyo, Japan). Activated carbon (coconut shell activated charcoal, KD-PWSP, average diameter 6.0 µm, BET surface area 1.324 × 10^3^ m^2^/g) was purchased from AS ONE Co., (Nishi-ku, Osaka, Japan). Phosphate-buffered saline (PBS) was purchased from FUJIFILM Wako Pure Chemical Co., Chou-ku, Osaka, Japan. Indole and Tryptophan were purchased from Tokyo Chemical Industry Co., Ltd., Chiyoda-ku, Tokyo, Japan, and used as received. Other reagents were purchased from Kanto Chemical Co. Inc. and used as received.

### 4.2. Preparation of the Activated-Carbon-Encapsulating HSBC Gel

**Figure 5:**
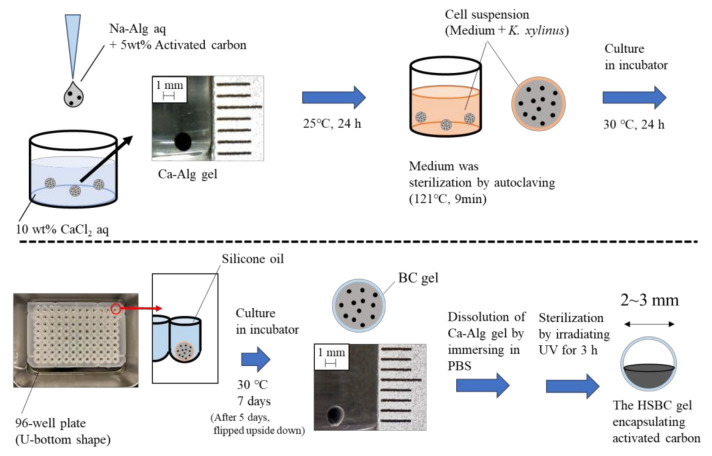
Preparation method for HSBC gel encapsulating activated carbon.

We synthesized an activated-carbon-encapsulating HSBC gel utilizing the methodology established in our previous research [33]. Figure 5 shows, in detail, the procedure for preparing the activated-carbon-encapsulating HSBC gel.

Spherical alginate gels (Ca–Alg gels) containing activated carbon were prepared using the method described below. Activated carbon (5 wt%) was suspended in 1 wt% sodium alginate aqueous solution (Na–Alg aq.); next, 5 µL of Na–Alg aq. was dropped into 10 wt% calcium chloride aqueous solution (CaCl_2_ aq.). The obtained Ca–Alg gels were washed via immersion in Milli-Q water.

The medium was sterilized by autoclaving (121 °C, 9 min), and then *Komagataeibacter xylinus* (*K. xylinus*, IFO13772) was cultured in the medium at 30 °C for 3 days. The spherical Ca–Alg gels that included the activated carbon were immersed in the cultured cell suspension and inoculated with *K. xylinus* for one day at 30 °C. The cell suspension remained at the Ca–Alg gel surface, and spherical Ca–Alg gels were immersed in each well of a U-shaped bottom 96-well plate filled (Caplugs Evergreen, item: 290-8353-03R) with silicone oil. While maintaining these states, *K. xylinus* in the cell suspension was cultured for 7 days at 30 °C. After the BC gelatinous membrane was biosynthesized, the obtained gel was impregnated in PBS and irradiated with UV light for 3 h to remove Ca-Alg gel spherical nuclei and sterilize *K. xylinus* to prepare activated-carbon-encapsulating HSBC gel.

The amount of encapsulated activated carbon was calculated as follows. The average dry weight of the HSBC gel without activated carbon was 32.0 μg, and the weight of the HSBC gel encapsulating activated carbon was 320.6 μg. Therefore, the average amount of activated carbon contained was 288.6 μg [33].

### 4.3. Stability Evaluation of ACEGs in Different pH Environments

#### 4.3.1. Stability Evaluation of ACEGs in Static Conditions

ACEG and 3 mL of test solution were placed in a PMMA cuvette with a volume of 4.5 mL and left undisturbed at room temperature for 48 h. To evaluate stability across different pH conditions, Milli-Q water, Disintegration Test Solution No. 1 (pH 1.2) and Solution No. 2 (pH 6.8), and NaOH aq (pH 12.6) were used as test solutions. Although a strongly basic environment does not exist in vivo, this condition was included to evaluate the stability of ACEGs under extreme basic environments.

#### 4.3.2. Stability Evaluation of ACEGs in Agitation Conditions

The permeability of the activated carbon dispersion decreased with an increase in the amount of activated carbon. If the activated carbon had leaked out of the HSBC gel in the test solution, the permeability of the solution would have decreased. Therefore, the leakage of activated carbon from the HSBC gel was evaluated by measuring its transmittance using UV-Vis spectroscopy. HSBC gels without activated carbon (10 samples) and ACEGs (10 samples) were placed in 30 mL of test solution, respectively, and stirred for 48 h at room temperature. The test solutions were used under the same conditions as in the static test (See Section 4.3.1). After 48 h, 2 mL of the measurement sample was taken from the stirred solution. The leakage of activated carbon was evaluated by measuring the transmittance at wavelengths from 300 to 800 nm using a UV-Vis spectrophotometer (V-530, JASCO Corporation, Tokyo, Japan). As a model for the leakage of the encapsulated activated carbon, 0.1 mg/mL activated carbon dispersion was also measured in the same manner.

In addition, the influence of the culture medium on the activated carbon was investigated. Activated carbon was immersed in the culture medium for 24 h and, subsequently, in NaOH aqueous solution for 48 h. The activated carbon was then removed using a syringe filter with a pore size of 0.2 µm, and the transmittance of the NaOH aqueous solution was measured.

### 4.4. Evaluation of Adsorption Capacity of Uremic Toxins in the Presence of Coexisting Ions

AST-120 (KREMEZIN^®^) adsorbs uremic toxins and their precursors, facilitating their excretion via stool. This study evaluated the adsorption of tryptophan and indole, precursors of uremic toxins, in activated carbon within a HSBC gel. Indole is produced by the catabolism of tryptophan by intestinal bacteria. Hence, the adsorption capacity of the ACEG was evaluated using Milli-Q water and Disintegration Test Solution No. 2, which has a pH range close to that of intestinal fluid. Disintegration Test Solution No. 2, as a model for intestinal fluid, has also been used in the Japanese Pharmacopoeia to evaluate enteric dissolving preparations.

Adsorption experiments were performed using indole or tryptophan as uremic toxin precursors at different concentrations. ACEGs were immersed in Milli-Q water within a small environmental test chamber at 70 °C for 3 h prior to testing. A 5.0 mL uremic toxin precursor solution and ACEG (1 sample) were added to each vessel and stirred inside a small environmental test chamber at 37 °C for 48 h on a plate shaker. The concentration of uremic toxin precursors before and after adsorption was quantified by absorbance at the maximum absorption wavelength (λ max indole: 270 nm; tryptophan: 280 nm), measured using a UV-Vis spectrophotometer (V-630, V-730, JASCO Corporation).

The equilibrium adsorption amount per unit mass of adsorbent *Q_e_* [µmol/mg] at each concentration was calculated using Equation (1):

(1) $$Q_{e}=\frac{\left(C_{i}-C_{e}\right)V}{m}$$

where *C_i_* and *C_e_* [µmol/mL] are the initial and equilibrium liquid phase concentrations of the uremic toxin precursor, respectively; *V* is the volume of solution [mL]; and m is the weight of the encapsulated activated carbon [mg].

Next, to evaluate the adsorption capacity, the saturated adsorption amount was calculated using Langmuir’s adsorption isotherm (2), which is as follows:

(2) $$Q_{e}=\frac{Q_{m}KC_{e}}{1+KC_{e}}$$

where *Q_e_* is the equilibrium adsorption amount [µmol/mg] corresponding to the complete monolayer coverage of the surface, *C_e_* is the adsorption equilibrium concentration [µmol/mL], and *K* is the Langmuir constant. *Q_m_* represents the practical limiting adsorption capacity (saturated adsorption amount) when the surface is completely covered with adsorbed molecules. The linear rearrangement of Equation (2) yields Equation (3):

(3) $$\frac{C_{e}}{Q_{e}}=\frac{C_{e}}{Q_{m}}+\frac{1}{KQ_{m}}$$

*Q_m_* was obtained from the slope of the *C_e_*/*Q_e_* vs. *C_e_* plot. The amount of activated carbon encapsulated in the HSBC gel was 289 µg, as previously reported [33].

## Figures and Tables

**Table 1:** *Q_m_*, Langmuir constant *K*, and *R*^2^ of ACEGs under each condition.

Adsorbent	Target Molecule	Solvent	*Q_m_*[µmol/mg]	*K*	*R* ^2^	
Activated carbon powder	Indole	Milli-Q	5.06	7.38	0.99	
Activated carbon encapsulated HSBC gel	Indole	Milli-Q	3.23	2.73	0.99	Figure 4a
	Indole	Disintegration test solution 2	2.62	4.38	0.99	Figure 4b
	Tryptophan	Milli-Q	3.60	18.64	0.99	Figure 4c
	Tryptophan	Disintegration test solution 2	1.32	6.86	0.97	Figure 4d

## Data Availability

Data is contained within the article.

## References

[1] Nitta K., Goto S., Masakane I., Hanafusa N., Taniguchi M., Hasegawa T., Nakai S., Wada A., Hamano T., Hoshino J. (2020). Annual dialysis data report for 2018, JSDT Renal Data Registry: Survey methods, facility data, incidence, prevalence, and mortality. Ren. Replace. Ther..

[2] Poznyak A.V., Sadykhov N.K., Kartuesov A.G., Borisov E.E., Sukhorukov V.N., Orekhov A.N. (2022). Atherosclerosis Specific Features in Chronic Kidney Disease (CKD). Biomedicines.

[3] Nigam S.K., Bush K.T. (2019). Uraemic syndrome of chronic kidney disease: Altered remote sensing and signalling. Nat. Rev. Nephrol..

[4] Wei J., Li R., Zhang P., Jin H., Zhang Z., Li Y., Chen Y. (2023). Efficient selective removal of uremic toxin precursor by olefin-linked covalent organic frameworks for nephropathy treatment. Nat. Commun..

[5] Summers C.S., Quimby M.J., Isaiah A., Suchodolski S.J., Lunghofer J.P., Gustafson L.D. (2019). The fecal microbiome and serum concentrations of indoxyl sulfate and p-cresol sulfate in cats with chronic kidney disease. J. Vet. Intern. Med..

[6] Ribeiro A., Liu F., Srebrzynski M., Rother S., Adamowicz K., Wadowska M., Steiger S., Anders H.-J., Schmaderer C., Koziel J. (2023). Uremic Toxin Indoxyl Sulfate Promotes Macrophage-Associated Low-Grade Inflammation and Epithelial Cell Senescence. Int. J. Mol. Sci..

[7] Ethical Drugs: KREMEZIN®. https://www.kegg.jp/medicus-bin/japic_med?japic_code=00067063.

[8] Green J.P., McCauley W. (2006). Bowel perforation after single-dose activated charcoal. Can. J. Emerg. Med..

[9] Goulbourne K.B., Cisek J.E. (1994). Small-Bowel Obstruction Secondary to Activated Charcoal and Adhesions. Ann. Emerg. Med..

[10] Dorrington C.L., Johnson D.W., Brant R., Multiple Dose Activated Charcoal Complication Study Group (2003). The frequency of complications associated with the use of multiple-dose activated charcoal. Ann. Emerg. Med..

[11] Abe I. (1999). Multifunctional Materials: Activated Carbon, Charcoal. J. Jpn. Soc. Colour Mater..

[12] Cao L., Li N. (2021). Activated-carbon-filled agarose hydrogel as a natural medium for seed germination and seedling growth. Int. J. Biol. Macromol..

[13] Lee J.W., Han J., Choi Y.-K., Park S., Lee S.H. (2023). Reswellable alginate/activated carbon/carboxymethyl cellulose hydrogel beads for ibuprofen adsorption from aqueous solutions. Int. J. Biol. Macromol..

[14] Hoshi T., Suzuki M., Ishikawa M., Endo M., Aoyagi T. (2019). Encapsulation of Micro- and Milli-Sized Particles with a Hollow-Type Spherical Bacterial Cellulose Gel via Particle-Preloaded Droplet Cultivation. Int. J. Mol. Sci..

[15] Klemm D., Heublein B., Fink H.-P., Bohn A. (2005). Cellulose: Fascinating Biopolymer and Sustainable Raw Material. Angew. Chem. Int. Ed..

[16] Fu C.-C., Hsiao Y.-S., Ke J.-W., Syu W.-L., Liu T.-Y., Liu S.-H., Juang R.-S. (2020). Adsorptive removal of p-cresol and creatinine from simulated serum using porous polyethersulfone mixed-matrix membranes. Sep. Purif. Technol..

[17] Juang R.-S., Li Y.-M., Hsiao Y.-S., Fu C.-C., Liu S.-H. (2024). Synergistic adsorption of creatinine and p-cresol from simulated serum using zeolites in electrospun fibrous mixed-matrix membranes. Sep. Purif. Technol..

[18] Cheng Y.-C., Fu C.-C., Hsiao Y.-S., Chien C.-C., Juang R.-S. (2018). Clearance of low molecular-weight uremic toxins p-cresol, creatinine, and urea from simulated serum by adsorption. J. Mol. Liq..

[19] Hsiao Y.-S., Tran H.N., Ke J.-W., Fu C.-C., Syu W.-L., Liu S.-H., Juang R.-S. (2022). Porous cellulose acetate mixed-matrix membrane adsorbents for efficient clearance of p-cresol and creatinine from synthetic serum. J. Taiwan Inst. Chem. Eng..

[20] Stiapis C.S., Skouras E.D., Pavlenko D., Stamatialis D., Burganos V.N. (2018). Evaluation of the Toxin-to-Protein Binding Rates during Hemodialysis Using Sorbent-Loaded Mixed-Matrix Membranes. Appl. Sci..

[21] Wang W., Wang Z., Li K., Liu Y., Xie D., Shan S., He L., Mei Y. (2023). Adsorption of uremic toxins using biochar for dialysate regeneration. Biomass Convers. Biorefinery.

[22] Guangle Q., Gan Z., Dapeng C., Jingjie S. (2024). Adsorption of uremic toxins by modified activated carbon of different mesh with sulfuric acid. Adsorption.

[23] Adler T.K., Albert A. (1963). The Biological and Physical Properties of the Azaindoles. J. Med. Chem..

[24] Lide D.R. (2005). Handbook of Chemistry and Physics.

[25] Fedotova M.V., Kruchinin S.E. (2014). Ion-binding of glycine zwitterion with inorganic ions in biologically relevant aqueous electrolyte solutions. Biophys. Chem..

[26] Fedotova M.V., Dmitrieva O.A. (2015). Ion-selective interactions of biologically relevant inorganic ions with alanine zwitterion: A 3D-RISM study. Amino Acids.

[27] Urano K., Nishimura Y., Yanaga Y. (1975). Adsorption of polyethylene glycol of different molecular weights on activated carbon in water. J. Chem. Soc. Jpn..

[28] Lorenc-Grabowska E. (2016). Effect of micropore size distribution on phenol adsorption on steam activated carbons. Adsorption.

[29] Jiang X.-Y., Li L., Yan J.-N., Wang C., Lai B., Wu H.-T. (2024). Binary hydrogels constructed from lotus rhizome starch and different types of carrageenan for dysphagia management: Nonlinear rheological behaviors and structural characteristics. Food Chem. X.

[30] Kulkarni S., Londhe V. (2021). Oral jelly of metformin hydrochloride—Formulation development using Design of Experiments and characterization. J. Drug Deliv. Sci. Technol..

[31] Choi S.M., Rao K.M., Zo S.M., Shin E.J., Han S.S. (2022). Bacterial Cellulose and Its Applications. Polymers.

[32] Roberts E.L., Abdollahi S., Oustadi F., Stephens E.D., Badv M. (2023). Bacterial-Nanocellulose-Based Biointerfaces and Biomimetic Constructs for Blood-Contacting Medical Applications. ACS Mater. Au.

[33] Hoshi T., Endo M., Hirai A., Suzuki M., Aoyagi T. (2020). Encapsulation of Activated Carbon into a Hollow-Type Spherical Bacterial Cellulose Gel and Its Indole-Adsorption Ability Aimed at Kidney Failure Treatment. Pharmaceutics.

